# Discovery of Known and Novel Viruses in Wild and Cultivated Blueberry in Florida through Viral Metagenomic Approaches

**DOI:** 10.3390/v13061165

**Published:** 2021-06-18

**Authors:** Norsazilawati Saad, James W. Olmstead, Arvind Varsani, Jane E. Polston, Jeffrey B. Jones, Svetlana Y. Folimonova, Philip F. Harmon

**Affiliations:** 1 Department of Plant Pathology, University of Florida, Gainesville, FL 32611, USA; jep@ufl.edu (J.E.P.); jbjones@ufl.edu (J.B.J.); svetlana@ufl.edu (S.Y.F.); 2 Department of Plant Protection, Faculty of Agriculture, Universiti Putra Malaysia, Serdang 43400, Malaysia; 3 Horticultural Sciences Department, University of Florida, Gainesville, FL 32611, USA; james.olmstead@driscolls.com; 4 The Biodesign Center of Fundamental and Applied Microbiomics, School of Life Sciences, Center for Evolution and Medicine, Arizona State University, 1001 S. McAllister Ave, Tempe, AZ 85287, USA; arvind.varsani@asu.edu; 5 Structural Biology Research Unit, Department of Integrative Biomedical Sciences, University of Cape Town, Cape Town 7925, South Africa

**Keywords:** metagenomics, viromes, blueberry, Florida

## Abstract

Southern highbush blueberry (interspecific hybrids of *Vaccinium corymbosum* L.) is cultivated near wild *V. corymbosum* as well as closely related species in Florida, USA. The expansion of blueberry cultivation into new areas in Florida and deployment of new cultivars containing viruses can potentially increase the diversity of viruses in wild and cultivated *V. corymbosum*. In this study, viral diversity in wild and cultivated blueberries (*V. corymbosum*) is described using a metagenomic approach. RNA viromes from *V. corymbosum* plants collected from six locations (two cultivated and four wild) in North Central Florida were generated by high throughput sequencing (HTS) and analyzed using a bioinformatic analysis pipeline. *De novo* assembled contigs obtained from viromes of both commercial and wild sites produced sequences with similarities to plant virus species from a diverse range of families (*Amalgaviridae*, *Caulimoviridae*, *Endornaviridae*, *Ophioviridae*, *Phenuiviridae*, *and* *Virgaviridae*). In addition, this study has enabled the identification of blueberry latent virus (BlLV) and blueberry mosaic associated ophiovirus (BlMaV) for the first time in Florida, as well as a tentative novel tepovirus (blueberry virus T) (BlVT) in blueberry. To the best of our knowledge, this is the first study that compares viral diversity in wild and cultivated blueberry using a metagenomic approach.

## 1. Introduction

The identification of new plant viruses using viral metagenomics has been described in numerous publications [1,2]. While the wild plant species in natural settings are known to potentially harbor uncharacterized viruses, most metagenomics studies have focused on the cultivated plant species [2]. The introduction of new crops to areas where they have never been grown before, as well as the implementation of intensive cropping systems, both of which lead to new encounters with virulent viruses infecting crops or indigenous plants, has facilitated the emergence of damaging virus epidemics around the world [3]. In Florida, the low-chill southern highbush blueberries (SHB) are cultivated near wild plants of the same and related species. The diverse communities of native *Vaccinium* spp. and the adjacent Florida’s blueberry production areas could serve as a reservoir for a diverse assemblage of viruses in these species, thus causing spillover and spillback between the wild and cultivated hosts [4]. Another pathway that can potentially cause new emerging viruses in cultivated *Vaccinium* spp. is the lack of virus screening prior to the use of native, wild blueberries in the development of new SHB cultivars.

Fifteen species of viruses have been recorded in highbush, lowbush, and rabbiteye blueberries around the world, including RNA and DNA viruses from eight known and two unassigned genera [5]. Only two virus species have been documented in Florida: blueberry necrotic ring blotch virus (BNRBV) (genus *Blunervirus*) [6] and blueberry red ringspot virus (BRRV) (genus *Soymovirus*) [7]. Since viral diseases are not currently a major threat to the Florida blueberry industry, no survey on the status of blueberry viruses has been performed. Therefore, this study with the following objectives was carried out to (1) characterize and compare the virus population of the wild and cultivated *Vaccinium corymbosum* in Florida using a metagenomic approach; and (2) identify and characterize novel and known viral sequences in the blueberry viromes. We showed that analysis of viromes determined from double-stranded RNA (dsRNA)-enriched samples using metagenomics is an excellent tool to explore viral diversity of RNA viruses of all genome types (dsRNA, positive- and negative-sense single-stranded RNA (ssRNA)). Metagenomic analysis of the RNA viromes has shown that viral diversity in the wild *V. corymbosum* is found to be greater than that in the cultivated species, with considerable overlap and unique genera represented in both species. This study has also led to the discovery of two known blueberry viruses, BlLV (genus *Amalgavirus*) and BlMaV (genus *Ophiovirus*), that are new to Florida, as well as a tentative novel tepovirus (BlVT) that has never been reported in blueberry. In addition, this research is the first to show that BlMaV can be found in wild highbush, *V. corymbosum.*

## 2. Materials and Methods

### 2.1. Plant Materials, Sample Preparation and Generation of V. corymbosum RNA Plant Viromes

**Figure 1:**
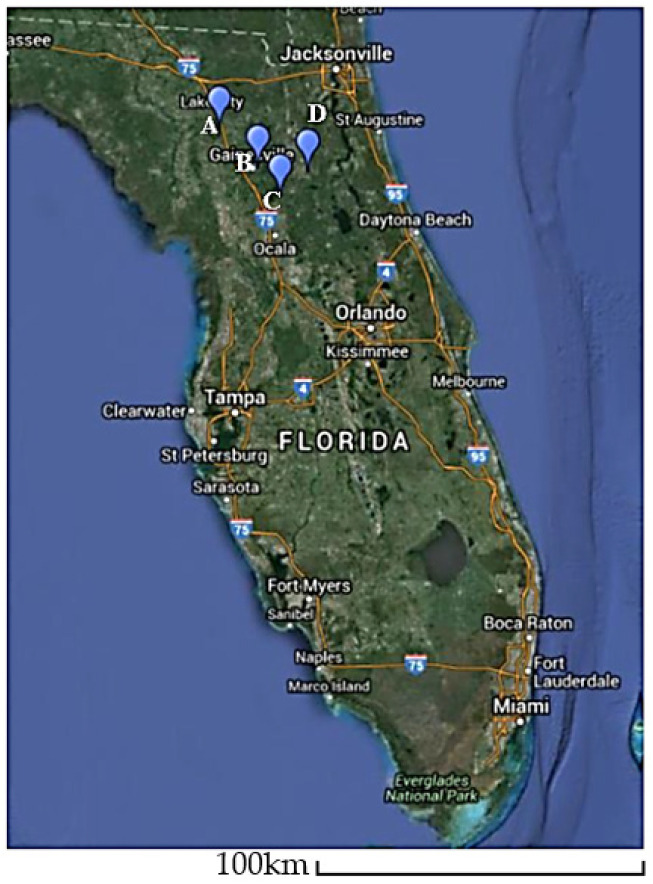
Map showing the locations of the collected blueberry samples. Adapted from [5]. (A) O’leno State Park, High Springs; (B) Morning Side Nature Center, Gainesville; (C) Island Grove; (D) Interlachen.

Twenty samples per site (*n* = 120) with and without virus-like symptoms were randomly collected from wild and cultivated blueberries (i.e., *V. corymbosum*) in central Florida. The wild blueberry samples were collected from four locations in Florida, USA: (1) O’leno State Park, High Springs, a wild site not adjacent to commercial blueberries; (2) Morning Side Nature Center, Gainesville, a wild site adjacent to residences; (3) Interlachen, a wild site neighboring commercial blueberry production; and (4) Island Grove, another wild site neighboring commercial blueberry production (Figure 1). The cultivated blueberries were collected from two commercial plantings in Interlachen and Island Grove, Florida, USA. All samples were collected during the northern hemisphere Fall of 2014 and 2015.

Total nucleic acid from leaf tissue of twenty *V. corymbosum* samples that were pooled by location was extracted and enriched for dsRNA following the previously published protocol [8]. Samples of dsRNA (100 ng) from each of the six locations were sequenced on the Illumina HiSeq 2500 platform (Macrogen, Inc., Seoul, Korea) by multiplexing to generate RNA libraries containing 150 bp paired-end sequences.

### 2.2. Analyses of RNA Plant Viromes

**Figure 2:**
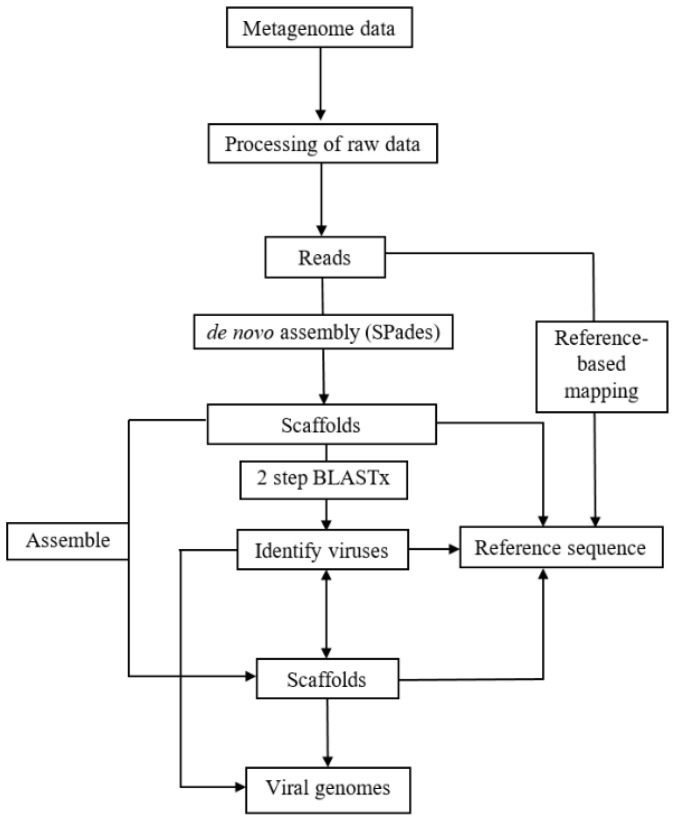
Virome analysis pipeline used for identification of viruses. Raw reads from metagenome data were processed by filtering the reads based on quality and trimming the adapter sequences. These reads were *de novo* assembled to produce contigs that were then subjected to a two-step BLASTx analysis to identify plant viruses, which include a comparison to a local plant virus database followed by comparison to a non-redundant GenBank protein database. Contigs with homology to the same virus species were then assembled to produce scaffolds. Apart from de novo assembly, a reference-based mapping approach was also used to obtain complete or partial viral genomes. Published viral genomes and scaffolds were used as reference sequences for known and novel viruses, respectively. Adapted from [5].

Reads from the RNA libraries were analyzed according to the virome analysis pipeline illustrated in Figure 2. Raw reads from each library were processed using Trimmomatic software to remove adapters and low-quality reads [9]. The quality-filtered reads were de novo assembled to produce contigs and scaffolds by using SPAdes version 3.7.0 assembler using k-mer 55, 77, and 99 [10]. Only scaffolds with length ≥500 nts were used for downstream analysis. These scaffolds were subjected to a two-step BLASTx [11] to identify plant viruses with the highest sequence match by comparing the scaffolds to a local plant virus protein database (http://virusdetect.feilab.net/cgi-bin/virusdetect/index.cgi) [12] (Accessed on 16 February 2017) followed by a non-redundant GenBank protein database using a threshold e-value of 10^−5^. Scaffolds with similarity to plant viruses were organized by family, genus, and species according to the 2016 Virus Taxonomy Release of the International Committee on Taxonomy of Viruses (ICTV) website (https://talk.ictvonline.org/taxonomy/) (Accessed on 10 March 2017) for approved virus species. Scaffolds with hits to virus species not yet approved by ICTV were assigned to its corresponding genus and family based on the information provided in the NCBI Taxonomy Database (https://www.ncbi.nlm.nih.gov/taxonomy) (Accessed on 25 April 2017). Scaffolds with sufficient genome coverage were directly used for genome analysis or subjected to another round of assembly using the Geneious assembler built in Geneious 9.1.6 to produce longer scaffolds. These scaffolds were mapped against the corresponding reference genome using Geneious mapper in Geneious 9.1.6 to observe the genome coverage. The scaffolds and the reference sequences were compared by alignment to predict the open reading frames (ORFs) and coding sequences (CDS). Reads were aligned to published viral sequences and scaffolds in a reference-based mapping approach using Bowtie2 to obtain complete viral genomes for known and novel viruses, respectively [13].

### 2.3. Sequence and Phylogenetic Analyses

Pairwise identity comparison between selected virus sequences obtained from this study and other selected members from the corresponding viral genera were performed using a Sequence Demarcation Tool 1.2 (SDT) [14]. Multiple sequence alignment of the respective ORF and segment of the RNA sequence of the corresponding viruses obtained from this study and selected published reference sequences were computed in MUSCLE [15]. Phylogenetic analysis of the nucleotide and amino acid (aa) sequences of selected ORF was performed by the neighbor joining method in MEGA (version 7.0) using bootstrap tests with 1000 replicates [16]. A phylogenetic tree was generated to infer the relationship between the known and new virus sequences to other published viral sequences from the corresponding viral genera.

### 2.4. Validation and Detection In Vitro of Novel Tepovirus

A set of primers enabling the amplification of the whole CP region of the de novo assembled complete genome of the putative novel tepovirus was designed based on the movement protein gene (*mp*) region, and the 3′ UTR was used to detect the virus in the RT-PCR using pooled dsRNA extracts of *V. corymbosum* (Table S1). First-strand cDNA was synthesized with ImProm-II reverse transcriptase (Promega, Madison, WI, USA) according to the manufacturer’s instructions. A total of 10 uL reaction mixture containing 180 ng total RNA and 1 µM Oligo (dT)_21v_ primer was first incubated at 70 °C for 10 min. This was followed by the addition of 10 uL solution containing a final concentration of 6 mM MgCl_2_, 1 mM dNTP mix, 20 U Rnasin, and 1 uL reverse transcriptase, incubated at 25 °C for 5 min, 42 °C for 1 h, and 70 °C for 15 min. PCR was then carried out by adding the cDNA template (2 uL) in a reaction mixture containing 2.5 mM MgCl_2_, 0.5 mM dNTP mix, 0.5 µM of each forward and reverse primer, and 0.625 U *Taq* DNA Polymerase (New England Biolabs, Ipswich, MA, USA) using the following cycling conditions: initial denaturation at 94 °C for 3 min, 35 cycles of 94 °C (30 s), 62 °C (45 s), and 72 °C (1 min 10 s), and a final extension at 72 °C for 10 min. The RT-PCR reaction was carried out in the Eppendorf AG 22,331 Hamburg Mastercycler (Eppendorf AG., Hamburg, Germany). Amplified fragments of the PCR products (~1 kB) were visualized on 0.8% agarose gel and used as an inset in cloning reaction with pGem-T easy cloning kit (Promega, USA). The clones were sequenced by the Sanger method at Eurofins MWG Operon LLC (Eurofins Scientific, Louisville, Kentucky, USA). The presence of the novel tepovirus in twenty *V. corymbosum* samples in five cultivars collected from Island Grove was screened using the same RT-PCR assay described above.

## 3. Results

### 3.1. General Analyses of the RNA Plant Viromes

The RNA viromes generated from wild and cultivated *V. corymbosum* contained approximately 183 and 69 million paired-end reads of 150 nt in length, respectively, following the processing of reads (Table 1). *De novo* assembly of the reads using SPades assembler produced 16,530 and 6151 scaffolds of >500 nt in length from the wild and cultivated *V. corymbosum* viromes, respectively. Comparison of the scaffolds to the local plant virus protein database followed by non-redundant GenBank protein database in two-step BLASTx analyses yielded a total of 227 (1%) and 43 (0.007%) putative plant virus scaffolds with matches to known viral sequences from the wild and cultivated *V. corymbosum*, respectively. The percentage of putative plant virus scaffolds with sequence similarity to known viruses was calculated from the total scaffolds in both wild and cultivated viromes varied between locations, ranging from 0.60 to 1.86%. The highest and lowest number of putative plant virus scaffolds obtained from the viromes of wild *V. corymbosum* were identified from Interlachen and Island Grove, respectively. The viromes of cultivated *V. corymbosum*, however, contained a similar number of putative plant virus scaffolds.

### 3.2. Diversity and Comparison of Viral Populations among the Viromes

**Figure 3:**
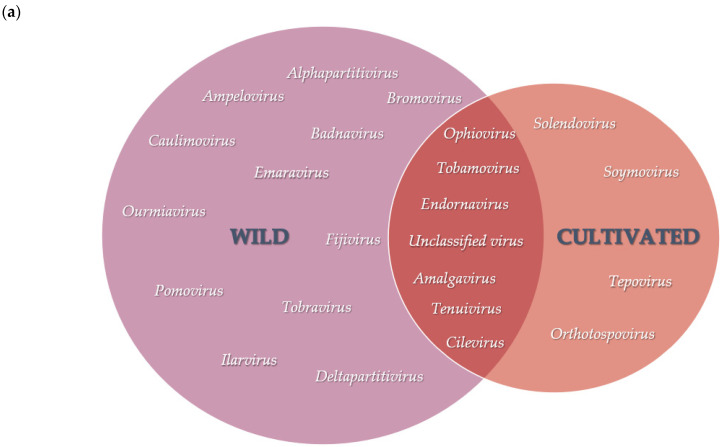

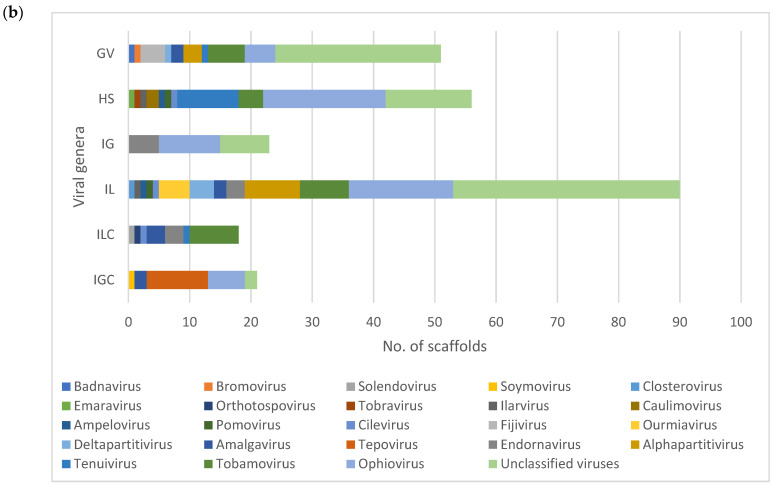
Viral populations in the RNA viromes of wild and cultivated *V. corymbosum*. (**a**) Diversity of virus sequences represented by the different range of viral genera of closely related viruses as identified by BLASTx analyses. The overlapping region indicates viral genera present in both sites. (**b**) Putative viral scaffolds with similarity to plant virus species from the different range of viral genera from each sampling location. GV, Gainesville; HS, High Springs; IL, Interlachen; IG, Island Grove; ILC, Interlachen cultivated site; IGC, Island Grove cultivated site. Unclassified viruses refer to species that have not been approved by the ICTV. Adapted from [5].

Scaffolds identified from both wild and cultivated *V. corymbosum* viromes produced sequence similarity to plant virus species from a total of 25 viral genera, representing a total of 17 viral families (Figure 3, Table S2). Of the viruses identified belonging to 23 viral genera, scaffolds with sequence similarities to virus species from 18 and 9 viral genera were identified in the wild and cultivated *V. corymbosum* viromes, respectively. Plant viruses from 6 viral genera belonging to the families *Amalgaviridae*, *Caulimoviridae*, *Endornaviridae*, *Ophioviridae*, *Phenuiviridae*, and *Virgaviridae* were identified in both wild and cultivated *V. corymbosum* viromes as shown in the overlapping Venn diagram (Figure 3a). Although there was considerable overlap observed between the wild and cultivated viromes, each had unique viral genera represented: 12 viral genera in the wild and 4 viral genera in the cultivated viromes. The viromes contained the greatest number of scaffolds with the highest sequence similarity to unclassified viruses (88), followed by ophioviruses (58) and tobamoviruses (26). In contrast, less than twelve scaffolds were recorded in each genus (Figure S1).

Viral populations among the viromes of wild and cultivated *V. corymbosum* in each location varied, as represented by the scaffolds with sequence similarity to virus species from different viral genera (Figure 3b). The wild *V. corymbosum* virome from Interlachen had the highest number of scaffolds with matches to plant virus sequences representing 13 viral genera, followed by High Spring (10), Gainesville (9), and Island Grove (2) (Figure 3b). The highest sequence similarity to unclassified viruses (86), followed by ophioviruses (52) and tobamoviruses (18) were obtained for the majority of scaffolds produced in the whole viromes of wild *V. corymbosum* (Figure S2). Each virome of wild *V. corymbosum* was found to be dominated by similar viral genera representing virus species closely related to the scaffolds. The *V. corymbosum* viromes from Gainesville and Interlachen were dominated by scaffolds that were closely related to unclassified viruses, whereas the other two locations were dominated by ophioviruses.

The virome of cultivated *V. corymbosum* from Interlachen compared to Island Grove produced a higher number of scaffolds with matches to plant virus sequences with 7 and 5 viral genera, respectively (Figure 3b). The highest sequence similarity to tepoviruses (10), followed by tobamoviruses (8), ophioviruses (6), and amalgaviruses (5) were obtained for the majority of scaffolds produced in the whole viromes of cultivated *V. corymbosum* (Figure S3). Of these viruses, the genera *Tobamovirus* and *Tepovirus* dominated the Interlachen and Island Grove viromes, respectively.

Viral populations between the viromes of *V. corymbosum* collected from the neighboring wild and cultivated sites at Interlachen and Island Grove were further compared. Scaffolds with the highest sequence similarity to virus species from five viral genera (*Amalgavirus*, *Cilevirus*, *Endornavirus,* and *Tobamovirus*) were identified in viromes of both wild and cultivated *V. corymbosum* collected from Interlachen. Scaffolds with the highest sequence similarity to unclassified viruses and ophioviruses were identified in viromes of both wild and cultivated *V. corymbosum* from Island Grove. The viromes of *V. corymbosum* collected from Interlachen at the wild site produced scaffolds with similarity to virus species from ten genera, while the cultivated site produced scaffolds with similarity to virus species from four genera. Similarly, viromes of *V. corymbosum* collected from Island Grove contained scaffolds closely related to virus sequences belonging to two viral genera exclusively identified in wild sites and five viral genera exclusively identified in cultivated sites.

### 3.3. Sequence Comparison and Phylogenetic Analyses of the Complete Viral Genomes

A total of ten complete genomes of five virus species, representing five viral genera, were assembled from the wild and cultivated *V. corymbosum* RNA viromes. The viruses were: blueberry latent virus (BlLV), *Amalgavirus*; blueberry mosaic associated ophiovirus (BlMaV), *Ophiovirus*; blueberry red ringspot virus (BRRV), *Soymovirus*; a putative new species closely related to prunus virus T (PrVT), *Tepovirus*; and tobacco mosaic virus (TMV), *Tobamovirus* (Table 2). As shown in Table 2, each virome contained at least one complete viral genome assembled *in silico*, except for the virome of wild *V. corymbosum* from Island Grove. In contrast, however, the virome of cultivated *V. corymbosum* from Island Grove yielded the maximum number of complete viral genomes compared to other viromes. Reads aligned to the various assembled genomes indicated the presence of single nucleotide polymorphisms (SNPs) in the corresponding virus sequences with varying frequencies (Table S3).

#### 3.3.1. Blueberry Latent Virus

The viromes of cultivated *V. corymbosum* from both Interlachen and Island Grove produced three (792–1116 nt in length) and two (1132–2401 nt in length) scaffolds with high sequence similarity to BlLV. Complete BlLV genome was further obtained by reference-based mapping approach using published reference genome (NC_014593). Mapping of reads from Interlachen to the reference genome showed that 258 (0.00062%) reads were aligned to the reference sequence. However, the Island Grove virome had 11× higher number of reads mapped to BlLV compared to those from Interlachen, with mapped reads of 1943 (0.007%). The average coverage of reads mapped to the reference genome was shown to be 10× for Interlachen and 75× for Island Grove. However, there were four missing nucleotides between nucleotide position 1066–1069 in the complete BlLV genome assembled from the virome of cultivated *V. corymbosum* at Interlachen.

The size of the genome assembled from the virome of cultivated *V. corymbosum* at Island Grove was 3432 nt, including the 5′ (167 nt) and 3′ (99 nt) UTRs. Similar to the genome organization of other published sequences of BlLV, the genome encodes for two ORFs that were partially overlapped by 314 nt [17]. A putative protein of 375 aa was encoded by ORF1 (1125 nt), while a putative fusion protein of 1054 aa was encoded by ORF2 (3162 nt). This contained the RNA-dependent RNA polymerase (RdRp) domain (aa 586–782) [17,18].

**Figure 4:**
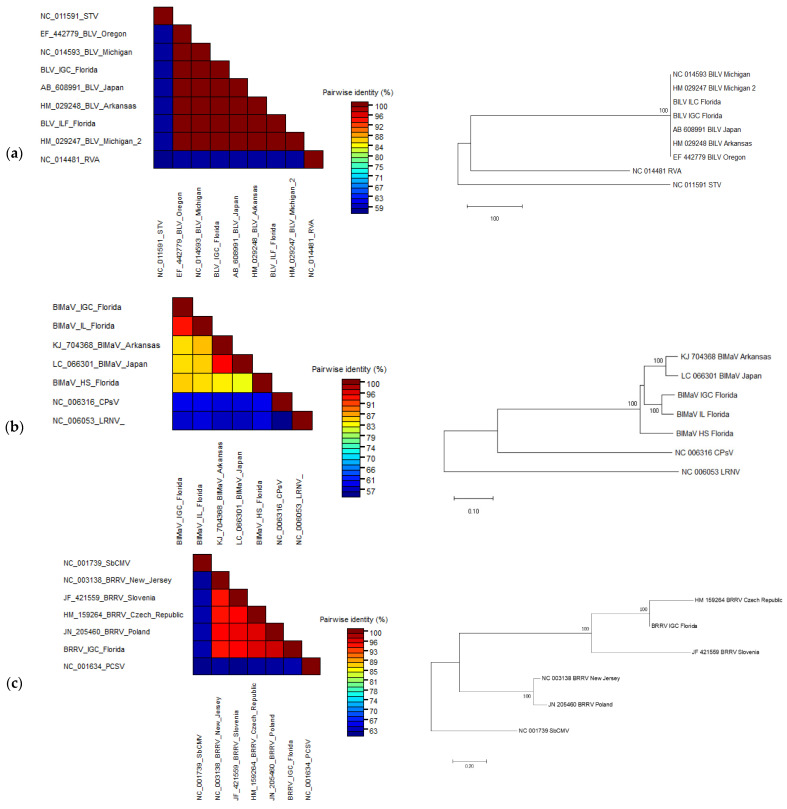

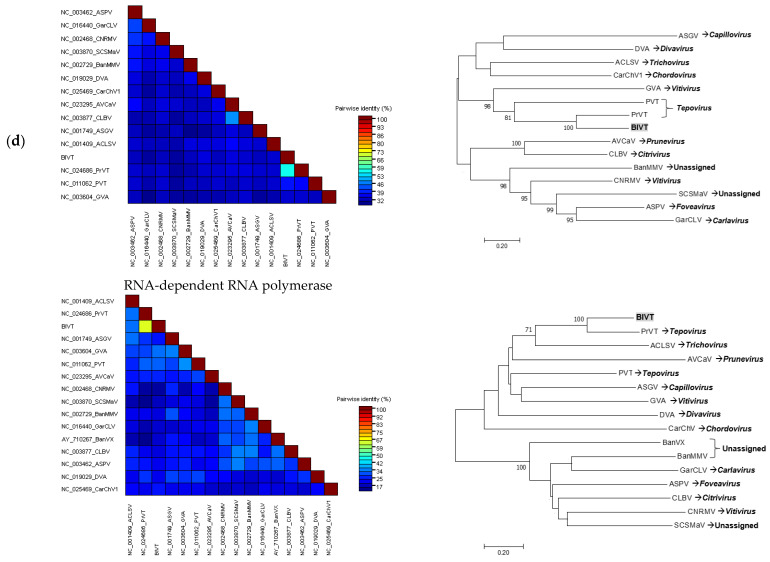
The pairwise comparison analysis in SDT and phylogenetic analysis in MEGA7 using nucleotide and amino acid sequences of different genome regions of the respective viruses. Pairwise sequence identities between the viruses are represented in different colors. The bootstrap consensus phylogenetic tree was constructed by neighbor-Joining using the Maximum Composite Likelihood and Poisson correction method for nucleotide and amino acid sequences, respectively, based on 1000 replicates, showing branch nodes more than 75% bootstrap values. (**a**) Pairwise identity and phylogenetic analysis using the full genome of BlLV isolates and selected members of the genus *Amalgavirus*. (**b**) Pairwise identity and phylogenetic analysis using the NP gene of BlMaV isolates and selected members of the genus *Ophiovirus*. (**c**) Pairwise identity and phylogenetic analysis using the full genome of BRRV isolates and selected members of the genus *Soymovirus*. (**d**) Pairwise identity and phylogenetic analysis using the RdRp and CP protein of the putative novel Tepovirus and selected members representing different genera in the family *Betaflexiviridae*. Accession numbers are shown in the figure. ACLSV, apple chlorotic leaf spot virus; ASGV, apple stem grooving virus; ASPV, apple stem pitting virus; AVCaV, apricot vein clearing associated virus; BanMMV, banana mild mosaic virus; BanVX, banana virus X; BBLV, blueberry latent virus; BlMaV, blueberry mosaic associated ophiovirus; BlVT, blueberry virus T; BRRV, blueberry red ringspot virus; CarChV1, carrot Ch virus 1; CLBV, citrus leaf blotch virus; CNRMV, cherry necrotic rusty mottle virus; CPsV, citrus psorosis virus; DVA—diuris virus A; GarCLV, garlic common latent virus; GVA, grapevine virus A; LRNV, lettuce ring necrosis virus; PCSV, peanut chlorotic streak virus; PrVT, prunus virus T; PVT, potato virus T; RVA, rhododendron virus A; SbCMV, soybean chlorotic mottle virus; SCSMaV, sugarcane striate mosaic-associated virus; STV, southern tomato virus. Adapted from [5].

Based on the pairwise nucleotide identity of the viral genomes as represented by the color-coded blocks, there were few nucleotide divergences observed between BlLV sequences obtained from viromes of cultivated *V. corymbosum* with other published sequences as shown by the high percentage of pairwise identity (>99%) (Figure 4a). Phylogenetic analysis of the deduced amino acid sequences using the whole genome of BlLV obtained from this study (Accession number: MN416031), and other published isolates showed that these sequences were clustered in the same clade with high confidence of bootstrap value (Figure 4a).

#### 3.3.2. Blueberry Mosaic Associated Ophiovirus

Six (1376–2429 nt in length) and twenty scaffolds (519–7946 nt in length) obtained from the viromes of *V. corymbosum* from Island Grove (cultivated) and High Springs, respectively, were used to assemble the three complete RNA segments of BlMaV. However, only the RNA3 segment was assembled from the virome of wild *V. corymbosum* from Interlachen. The percentage and average coverage of reads in the viromes of Island Grove and High Springs that were mapped to each assembled BlMaV RNA segment is shown in Table 3. The virome of High Spring produced a higher percentage of reads mapped to RNA1, while the Island Grove virome had a higher percentage of reads mapped to RNA2 and RNA3 (Table 3).

The length of each RNA segment assembled from each virome varied, although the size of corresponding ORFs were similar when compared to the reference sequence (Table 4). The complete segmented genome of BlMaV assembled from Island Grove and High Springs viromes accounted for 11,271 nt and 11,487 nt, respectively, and differed by 196 and 20 nt from the reference genomes (KJ704366–KJ704368) [19]. The complete genome consisted of RNA 1, RNA 2, and RNA 3 encoded for RdRp/23kDa proteins, movement proteins (MP), and nucleocapsid proteins (NP), respectively, which are similar in size with those of the reference genome, whereas the 5′ and 3′ untranslated regions (UTRs) of these RNA segments differed from the reference sequence. The complete ORF sequences of RdRp, MP, and NP assembled from the virome of Island Grove (Accession number: MZ055455–MZ055457) as well as NP assembled from the virome of High Springs and Interlachen (Accession number: MZ227013 and MZ227014) were deposited into the GenBank.

Each RNA sequence obtained from the virome was compared to the reference sequence (KJ704366–KJ704368) by pairwise local alignment. RNA1, 2, and 3 produced approximately between 80–81, 80, and 83% of nucleotide identity, respectively (Table 4). Pairwise nucleotide analysis was performed in SDT using the NP ORF sequence obtained from the viromes of Island Grove, High Springs, and Interlachen. As shown in Figure 4b, the NP obtained from Island Grove and Interlachen viromes shared the highest pairwise nucleotide identity of more than 90% and both sequences shared around 86% pairwise identity with sequence isolates from Arkansas (KJ704368) and Japan (LC066301). However, NP sequence assembled from High Springs virome appeared to be considerably diverged from other isolates, including those from Florida, by sharing just ~83% pairwise identity. Furthermore, the results of pairwise analysis using SDT were reflected in the phylogenetic analysis of the deduced NP aa sequences, whereby isolates from Arkansas and Japan were grouped in the same clade as those isolates from Island Grove and Interlachen with high confidence of bootstrap value, although both formed a separate subgroup (Figure 4b). As expected, the NP sequence from High Springs formed a distinct clade from other isolates.

#### 3.3.3. Blueberry Red Ringspot Virus

A single scaffold of 8392 nt in length was assembled from the virome of the Island Grove cultivated site, representing the complete genome of BRRV (Accession number: MZ004932). The percentage and average coverage of reads mapped to the BRRV scaffold in Bowtie2 were 0.95% and 4500×, respectively. Similar to the genome organization of other published sequences, the scaffold of BRRV encoding for eight ORFs: ORF I (movement protein), A, B, C, IV (coat protein), V (reverse transcriptase), VI (translational transactivator), and VII. Though, there were some differences in the length of ORFs I, C, RT, and VII, as well as the length of the whole-genome, which was the longest when compared to other isolates (Table 5).

Whole-genome pairwise comparison using SDT showed that the BRRV scaffold shared the highest nucleotide identity (97%) to the published sequence of BRRV isolates from the Czech Republic (HM159264) and Poland (JN205460) (Figure 4c). In addition, phylogenetic analysis using the whole genome of different BRRV isolates indicated that the BRRV genome recovered from this study was grouped in the same clade with isolates from the Czech Republic and Slovenia, while the isolate from New Jersey was grouped with the isolate from Poland (Figure 4c).

#### 3.3.4. Tentative New Species in the Genus Tepovirus

Apart from finding known viruses discussed so far, a total of 10 scaffolds, with the greatest length of 2504 nt and represented by 0.04% of the total mapped reads (225× average coverage), from the virome of the Island Grove cultivated site had the closest sequence similarity to PrVT in the BLASTx analyses. Mapping of these scaffolds against the reference sequence PrVT isolate Aze239 (NC_024686) [20], which was 6835 nt in length by Geneious assembler, showed that all 10 scaffolds spanned the whole genome of PrVT, including the 5′ and 3′ UTRs. The scaffolds were de novo assembled using Geneious assembler to produce larger scaffold of 7200 nt in length. Reads were mapped against the 7200 nt scaffold, which produced consensus sequences containing mixed bases of Y (C/T) and R (A/G) with a frequency of 0.2 and 0.3%, respectively. The ORFs of the draft genome were predicted in Geneious by sequence comparison to the PrVT genome.

Primers were designed in the movement protein (MP) ORF and 3′UTR regions of the draft genome to obtain the whole CP for validation and genome completion of the new virus by PCR. The sequence from the cloned fragment (1010 bp) showed more than 96% pairwise identity to the viral genome draft. Alignment of sequence obtained by the Sanger method to the draft genome allowed us to assemble the complete CP sequence of the new virus species, tentatively named as blueberry virus T (BlVT) (Accession number: MZ054240). This virus had genome organization similar to the previously described sequences of members from the genus *Tepovirus* in the *Betaflexiviridae*. The genome of 7200 nt contained three overlapping ORFs encoding for the RNA-dependent RNA polymerase (RdRp) (5457 nt), MP (1146 nt), and CP (663 nt). The genome also contained the 5′ (109 nt) and 3′ (264 nt) UTRs. The RdRp and MP region overlapped by 89 nt (bases 5463 to 5566), whereas the MP and CP overlapped by 350 nt (bases 6274 to 6623). The differences between the length of BlVT and PrVT in the RdRp, MP, and CP regions were 120, 6, and 3 nt, respectively (Table 6).

**Figure 5:**
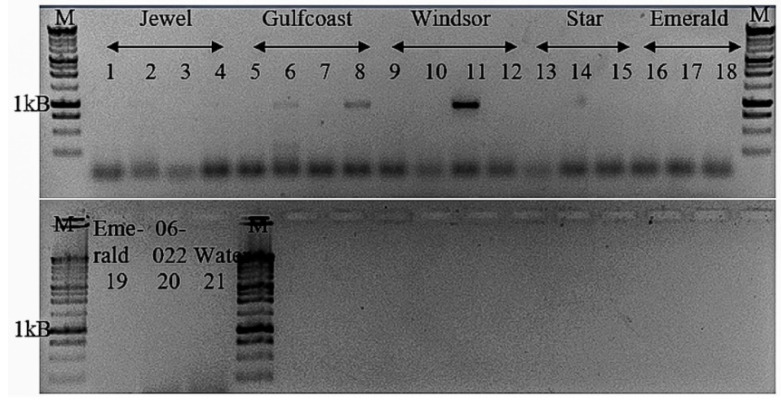
Validation of the putative new tepovirus in extracted RNA of different *V. corymbosum* cultivars from Island Grove by PCR, producing expected amplicons at ~1 kB. Cultivar’s name was shown at the top of the gel. M, 1 kB DNA ladder.

Pairwise nucleotide comparison between each ORF of BlVT and PrVT by MUSCLE indicated that these viruses shared between 59–70% pairwise identity in the corresponding ORFs and UTRs (Table 6). Pairwise amino acid comparison of the putative RdRp and CP encoded by BlVT with selected sequences representing members of the family *Betaflexiviridae* using SDT indicated that BlVT shared the highest identity to the RdRp (55%) and CP (64%) of PrVT (Figure 4d). Furthermore, phylogenetic analysis of the putative RdRp encoded by BlVT showed that this virus is grouped by highly significant bootstrap value with the same clade as PrVT and potato virus T (PVT), the only members of the genus *Tepovirus* (Figure 4d). While phylogenetic analysis of the putative CP encoded by BlVT again indicated that this virus was clustered with PrVT, both viruses however were separated from PVT. Additionally, BlVT was detected in 3 of the 20 samples collected from Island Grove. Two samples from the cultivar “Gulf Coast” and one sample from the cultivar “Windsor” tested positive for BlVT (Figure 5).

## 4. Discussion

Without doubt, plant viral metagenomics has contributed to our understanding of viral populations, as well as unravelling the etiology of viral diseases in various plant species. This is supported by the exponentially increased discovery of novel plant viruses that have been thoroughly described [1,2]. In this study, viral populations in the wild and cultivated species of *V. corymbosum*, collected from different locations in Florida, were characterized using a viral metagenomic approach. Less than 2% of the de novo assembled scaffolds obtained from all the RNA viromes were considered as putative plant virus scaffolds because the majority of scaffolds produced sequence similarity to either insect or fungal viruses (data not shown). The presence of scaffolds with the highest sequence match to viruses from a total of 23 genera indicated that the population of viruses in this host are greatly diverse. This is in agreement with the findings of diverse viral species in previous studies of various wild plant species through viral metagenomics [21,22,23,24,25]. A greater virus diversity was further observed in the viral population of wild *V. corymbosum* native to Florida, as shown by the number of viral genera, which is almost twice more than those of cultivated viromes. Several conditions could contribute to the greater diversity of viruses in the viral population of the wild *V. corymbosum* compared to the cultivated ones. These conditions include the presence of different wild *Vaccinium* species and other plant communities within and surrounding the areas, the occurrence of vectors, and abiotic factors at these locations. In addition, the presence of diverse plant communities within and surrounding the wild *V. corymbosum* may act as a virus reservoir, thus increasing the heterogeneity in the population of viruses due to their movement between these plants. The occurrence of insects as virus vectors in the wild area may be another important factor that contributes to the greater virus diversity in the wild *V. corymbosum* by facilitating the movement of viruses between plants. On the other hand, the movement of viruses in the cultivated plants may be restricted due to the implementation of agricultural management practices, which could contribute to the lower number of viral genera in this host.

Metagenomic analysis of the viromes of wild and cultivated species of *V. corymbosum* in Florida has enabled the reconstruction of ten consensus sequences of five virus species, including BlLV, BlMaV, BRRV, TMV, and a putative new species closely related to PrVT. Although the complete genome of TMV was detected in four out of six viromes, TMV was not included for further sequences analysis since it is well known that tobamoviruses are frequently found in various environments due to their high stability [26]. In addition, the presence of varying frequency of SNPs from the mapping of reads to the assembled viral genomes in the corresponding viromes suggests the possible presence of virus variants in the pooled samples. Although the assembled viral sequences could represent a consensus of various genomes, this does not affect the overall findings of this study, in which the metagenomic approach has led to the detection of several known and new viruses in wild and cultivated blueberry in Florida.

The complete genomes of BlLV, an amalgavirus, were recovered from viromes of cultivated *V. corymbosum*. Pairwise sequence comparison between the whole genome of BlLV from Florida with other published isolates showed more than 99% nucleotide identity. This finding was expected since it was previously reported that BlLV has a very stable population structure, with less than 0.5% diversity among partial and complete sequences of isolates from Japan and the US [17,27,28]. Phylogenetic analysis of the complete genome of BlLV isolates from Arkansas, Florida, Michigan, Oregon showed that these isolates clustered together, suggesting that the complete genome of BlLV recovered from Florida is another isolate of BlLV.

Scaffolds with high homology (96–97%) to an ophiovirus, BlMaV, were identified in all viromes except the cultivated *V. corymbosum* virome at Interlachen. The presence of scaffolds closely related to BlMaV in all viromes of wild *V. corymbosum* suggests that the occurrence of this virus in wild blueberry might be common. Pairwise comparison using the NP regions of BlMaV isolates assembled from this study (Island Grove, Interlachen, and High Springs) and those from Arkansas and Japan indicated that the isolate from Island Grove and Interlachen had 14% sequence divergence from the rest of the isolates. This result was supported by a previous study which found that BlMaV had low genetic diversity among isolates, as shown by 13% nt divergence in the NP regions [29]. In contrast, the isolate from High Springs showed up to ~17% nt divergent with other isolates, thus suggesting that this sequence might have evolved from reassortment between BlMaV variants. In addition, phylogenetic analysis showed that the High Springs isolate form a separate branch from those of Island Grove and Interlachen, suggesting that BlMaV could have been established earlier in the wild *V. corymbosum* at High Springs in order for diversification to occur.

The analysis of the RNA virome additionally allowed for the assembly of a complete BRRV genome, a dsDNA virus, from the virome of cultivated *V. corymbosum* at Island Grove. The detection of BRRV in the RNA virome is not surprising since it produces RNA/DNA intermediate during its replication cycle, thus making it possible to form dsRNA complex at some point. The genome organization and ORFs of BRRV isolate from Island Grove are similar to the recently published BRRV genomes identified from the cultivar “Emerald” in Florida [7]. Overall, whole-genome pairwise comparison analysis of the BRRV isolates from the Czech Republic, Island Grove, New Jersey, Poland, and Slovenia showed that these isolates had less than 10% nt divergence, implying low genetic diversity and thus suggesting that this virus has a very stable population structure. In addition, pairwise comparison and phylogenetic analysis indicated that BRRV isolate from Island Grove was closely related to an isolate from the Czech Republic, suggesting that there may be an exchange of BRRV-infected material between these regions.

In addition to the detection of known viruses of blueberry that are new to Florida, this study also led to the discovery of a novel virus that is closely related to PrVT, a member in the genus *Tepovirus* belonging to the family *Betaflexiviridae*. Partial resequencing completely validated the CP region of the de novo assembled scaffold obtained by metagenome analysis. Pairwise comparison of the RdRp and CP of the putative novel virus with the corresponding proteins of selected members representing different genera in the family *Betaflexiviridae* demonstrated that this novel virus was distantly related to other members of the respective viral family, though higher sequence homology was observed with PrVT. Furthermore, pairwise nucleotide comparison showed that the putative novel virus only shared 61 and 65% identity with the RdRp and CP of PrVT, respectively, which fall well below the currently accepted species demarcation criteria in the genus *Tepovirus*, described as having less than 72% nt identity between the RdRp and CP genes [30]. Phylogenetic analyses of these genes demonstrated that the novel virus is consistently grouped with PrVT. The similar size and organization of the new viral genome to other members of the family *Betaflexiviridae* as well as statistically supported phylogenetic grouping further suggested that this novel virus, proposed as BlVT, should be considered as a new species in the genus *Tepovirus*. The biological information regarding BlVT is still lacking due to the unknown vector as well as the limited knowledge on the spread of this virus in the field. Although molecular screening of BlVT in twenty *V. corymbosum* samples collected from Island Grove, Florida, indicated a 15% virus incidence, the infection of BlVT still could not be associated with specific virus symptoms at this point due to mixed viral infections in the plants. Hence, the primers developed in this study could potentially be used for detection of BlVT in blueberry or related crops in the future.

## Figures and Tables

**Table 1:** The number of processed reads and scaffolds, and the percentage (%) of putative plant virus scaffolds for each RNA library corresponding to different sampling sites. Adapted from [5].

Libraries	No. of Reads		No. of Scaffolds ≥500	No. of Putative Plant Virus Scaffolds	% of Putative Plant Virus Scaffolds
	Raw	Processed			
GV	52,166,912	48,198,079	4256	52	1.22
HS	50,757,234	46,322,605	3485	60	1.72
IL	44,181,866	41,424,158	4890	91	1.86
IG	50,931,730	47,527,410	3899	24	0.62
ILC	44,632,114	41,527,161	3361	20	0.60
IGC	30,729,018	27,504,778	2790	23	0.82

GV, Gainesville; HS, High Springs; IL, Interlachen; IG, Island Grove; ILC, Interlachen cultivated site; IGC, Island Grove cultivated site.

**Table 2:** The consensus sequence of complete viral genomes assembled from each plant virome using a virome analysis pipeline. The tick mark indicated the complete viral genomes that were successfully assembled from each RNA virome. Adapted from [5].

Virus	Genus	Vector	Transmission	GV	HS	IL	IG	ILC	IGC
BlLV	*Amalgavirus*	No	Transmitted through seed					✓	✓
BlMaV	*Ophiovirus*	UK	UK		✓				✓
BRRV	*Soymovirus*	UK	Vegetative propagation						✓
BlVT	*Tepovirus*	UK	UK						✓
TMV	*Tobamovirus*	No	Mechanical	✓	✓	✓		✓	

BlMaV, blueberry mosaic associated ophiovirus; BlLV, blueberry latent virus; BRRV, blueberry red ringspot virus; BlVT, blueberry virus T; TMV, tobacco mosaic virus; UK, unknown vector; GV, Gainesville; HS, High Springs; IG, Island Grove; IL, Interlachen; ILC, Interlachen cultivated site; IGC, Island Grove cultivated site.

**Table 3:** The percentage (%) and average coverage of mapped reads to each RNA segment of BlMaV obtained from virome of cultivated *V. corymbosum* from Island Grove and wild *V. corymbosum* from High Springs and Interlachen, and the percentage of pairwise nt identity between each RNA to the corresponding reference sequences. Adapted from [5].

RNA Segments	% of Mapped Reads (Average Coverage)			% Pairwise nt Identity to Ref. Seq.		
	IGC	HS	IL	IGC	HS	IL
1	0.01 (47×)	0.013 (102×)	-	81.2	79.9	-
2	0.004 (83×)	0.0005 (16×)	-	79.5	79.7	-
3	0.003 (61×)	0.007 (252×)	0.01 (368x)	83.4	82.7	84.6

Reference sequence (Ref. seq.): Accession number: KJ_04366-8; IGC, cultivated *V. corymbosum* from Island Grove; HS, High Springs; IL, wild *V. corymbosum* from Interlachen; k, kilo.

**Table 4:** The length of each RNA segment and the encoded ORFs of the reference sequence and BlMaV obtained from virome of cultivated *V. corymbosum* from Island Grove and wild *V. corymbosum* from High Springs and Interlachen. Adapted from [5].

RNA Segments	ORFs	Length of RNA (nt)				Length of ORFs (nt)				5′/3′ UTRs			
		RS	IGC	HS	IL	RS	IGC	HS	IL	RS	IGC	HS	IL
1	RdRp/23 kDa	7963	7747	7946	-	7014/585	7014/585	7014/576	-	238/19	-/25	190/43	-
2	MP	1934	1981	1939	-	1548	1545	1545	-	317/69	334/60	337/99	-
3	NP	1570	1543	1602	1591	1368	1368	1368	1368	128/77	111/64	165/127	163/61

Reference sequence (RS), Accession number: KJ704366-KJ704368; IGC, cultivated *V. corymbosum* from Island Grove; HS, High Springs; IL, wild *V. corymbosum* from Interlachen; k, kilo.

**Table 5:** Nucleotide length of each ORFs in different BRRV isolates from Florida and other regions. Adapted from [5].

Isolates	ORFs								Total Length
	I (MP)	A	B	C	IV (CP)	V (RT)	VI (TA)	VII	
CZ	1101	312	561	600	1488	2004	1284	462	8302
IGC-FL	1197	369	561	597	1488	2007	1284	522	8392
NJ	939	369	561	600	1461	1974	1287	429	8303
PL	939	369	561	594	1455	1974	1284	462	8265
SL	1110	369	561	588	1476	2043	1284	462	8299

CZ, Czech Republic; IGC-FL, Island Grove, Florida; NJ, New Jersey; PL, Polish; SL, Slovenia.

**Table 6:** The nucleotide length and pairwise nucleotide comparison of each ORF and the UTR of BlVT and PrVT (NC_024686). Adapted from [5].

Type of Region	Region	Length (nt)		% Pairwise nt Identity
		PrVT	BlVT	
ORFs	RdRp	5337	5457	61
	MP	1152	1146	70
	CP	666	663	65
UTRs	5′	46	109	68
	3′	79	264	59

## Data Availability

Data is contained within the article.

## Supplementary Materials

The following are available online at https://www.mdpi.com/article/10.3390/v13061165/s1, Table S1: Primer sequence designed based on the de novo assembled complete genome of a putative novel Tepovirus; Table S2: Plant viruses with its corresponding viral genera which produced closest sequence similarity to the scaffolds (>500 nt in length) as identified by BLASTx analyses of wild and cultivated *V. corymbosum* viromes; Table S3. The number of SNPs identified from reads aligned to the assembled viral genomes in the corresponding viromes using FreeBayes in Geneious Prime v2019.1.3 with default parameter; Figure S1. Total no. of identified plant virus scaffolds according to genera in the whole viromes of wild and cultivated *V. corymbosum* viromes; Figure S2. Total no. of identified plant virus scaffolds according to genera in the viromes of wild *V. corymbosum* viromes; Figure S3. Total no. of identified plant virus scaffolds according to genera in the viromes of cultivated *V. corymbosum* viromes.
