# Supplementary material for: Discovery of Known and Novel Viruses in Wild and Cultivated Blueberry in Florida through Viral Metagenomic Approaches
Source: Viruses. 2021 Jun 18;13(6):1165. doi: 10.3390/v13061165 (PMC8234961; doi:10.3390/v13061165)

Supplementary files

Table S1. Primer sequence designed based on the *de novo* assembled complete genome of a putative novel *Tepovirus*.

**Primer name**	**Length**	**Sequence**	**Binding region**	**Position in scaffold**
NT_F	20	AGGGGTGCGAATTTTAGGCA	MP	5,967- 5,986
NT_R	24	AACTAGACGAGGCTCTGGTG	3′UTR	6,976- 6,957

Table S2. Plant viruses with its corresponding viral genera which produced closest sequence similarity to the scaffolds (>500 nt in length) as identified by BLASTx analyses of wild and cultivated *V. corymbosum* viromes.

Closely related virus sp.	Genera	No. of hits	Max Scaffold length (nt)	Max Query cov (%)	Highest E-value	Highest Identity (%)	Closely matched proteins
**Gainesville**							
Tobacco mosaic virus	*Tobamovirus*	5	2687	100	0	100	MP, RdRp
Blueberry mosaic associated virus	*Ophiovirus*	5	2244	94	0	96	23kDa, RdRp
Raphanus sativus cryptic virus 1	Unclassified virus	5	1681	100	2.16E-168	65	CP, RdRp
Persimmon latent virus	Unclassified virus	16	6997	100	0	54	RdRp, PArp
Vicia faba partitivirus 1	Unclassified virus	1	908	96	4.82E-88	49	RdRp
Grapevine partitivirus	Unclassified virus	1	1066	84	1.19E-80	48	RdRp
Rose partitivirus	*Alphapartiti-virus*	3	1655	86	3.09E-107	42	CP, RdRp
Cassia yellow blotch virus	*Bromovirus*	1	577	86	1.02E-33	40	RdRp
Raphanus sativus cryptic virus 2	Unclassified virus	4	1239	93	2.63E-74	40	RdRp
Rice grassy stunt virus	*Tenuivirus*	1	518	90	9.66E-19	36	RdRp

Frangipani mosaic virus	*Tobamovirus*	1	517	80	1.57E-14	36	RdRp
Pepper cryptic virus 1	*Deltapartiti-virus*	1	672	60	1.09E-15	33	RdRp
Ambrosia asymptomatic virus 2 UKM-2007	*Badnavirus*	1	631	82	1.49E-18	31	RT
Southern tomato virus	*Amalgavirus*	1	850	64	9.69E-11	29	FP
Rice black streaked dwarf virus	*Fijivirus*	3	4333	81	1.18E-75	28	RdRp
Blueberry latent virus	*Amalgavirus*	1	680	60	6.60E-09	27	FP
Maize rough dwarf virus	*Fijivirus*	1	2742	61	3.83E-06	20	P2
**High Springs**							
Tobacco mosaic virus	*Tobamovirus*	4	3514	97	0	100	CP, RdRp
Blueberry mosaic associated virus	*Ophiovirus*	20	7946	100	0	98	23kDa, MP, NP, RdRp
Grapevine partitivirus	Unclassified virus	2	812	100	4.58E-53	77	RdRp
Persimmon latent virus	Unclassified virus	6	6476	99	0	53	PArp, RdRp
Figwort mosaic virus	*Caulimovirus*	1	1270	44	1.23E-83	48	RT
Raphanus sativus cryptic virus 1	Unclassified virus	1	975	73	1.38E-59	45	RdRp
Diuris penduncculata cryptic virus	Unclassified virus	1	745	97	3.20E-72	43	RdRp
Pinus sylvestris partitivirus NL-2005	Unclassified virus	2	602	89	3.74E-32	42	RdRp
Broad bean necrosis virus	*Pomovirus*	1	875	43	1.47E-20	39	RdRp
Citrus leprosis virus C	*Cilevirus*	1	855	71	4.62E-32	38	RdRp

Tobacco rattle virus	*Tobravirus*	1	1008	50	3.37E-24	36	RdRp
Strawberry vein banding virus	*Caulimovirus*	1	519	38	1.27E-06	35	RT
Rice grassy stunt virus	*Tenuivirus*	5	7978	92	1.22E-82	33	P1.339K, RdRp
Rose rosette virus	*Emaravirus*	1	506	49	7.83E-08	32	RdRp
Rice stripe virus	*Tenuivirus*	5	1403	94	2.24E-25	31	RdRp
Pineapple mealybug wilt-associated virus 3	*Ampelovirus*	1	1020	62	5.63E-19	30	RdRp
Yerba mate endornavirus 1	Unclassified virus	1	794	48	7.32E-05	29	PP
Vicia faba partitivirus 1	Unclassified virus	1	543	83	4.24E-13	27	RdRp
Tobacco streak virus	*Ilarvirus*	1	645	80	4.64E-08	23	RdRp
**Interlachen**							
Tobacco mosaic virus	*Tobamovirus*	8	1316	100	0	100	CP, MP, RdRp
Blueberry mosaic associated virus	*Ophiovirus*	17	3458	100	0	97	23kDa, MP, NP, RdRp
Grapevine partitivirus	Unclassified virus	5	1792	100	6.90E-101	80	RdRp
Diuris pendunculata cryptic virus	Unclassified virus	3	1874	100	1.19E-85	74	CP, PP (RdRp)
Rose partitivirus	Unclassified virus	2	1867	92	2.13E-161	65	RdRp
Raphanus sativus cryptic virus 1	Unclassified virus	9	2009	100	1.95E-143	49	CP, RdRp
Radish partitivirus JC-2004	Unclassified virus	1	523	46	6.33E-18	47	RdRp

Supplementary files

Persimmon latent virus	Unclassified virus	2	798	98	2.04E-64	46	RdRp
Persimmon cryptic virus	Unclassified virus	2	1316	82	3.18E-47	45	RdRp
Raphanus sativus cryptic virus 2	Unclassified virus	4	1578	90	1.53E-54	43	RdRp
Pinus sylvestris partitivirus NL-2005	Unclassified virus	4	933	99	1.31E-38	42	RdRp
Vicia faba partitivirus 1	Unclassified virus	1	908	93	8.44E-71	42	RdRp
Arhar cryptic virus-I	Unclassified virus	1	1243	87	1.58E-66	40	RdRp
Beet cryptic virus 1	*Alphapartiti-virus*	4	789	88	6.06E-46	38	RdRp
Beet cryptic virus 1	*Alphapartiti-virus*	4	789	88	6.06E-46	38	RdRp
Vicia cryptic virus	*Alphapartiti-virus*	1	551	69	3.72E-21	38	RdRp
Pepper cryptic virus 1	*Deltapartiti-virus*	2	1364	99	1.93E-79	38	RdRp
Black raspberry cryptic virus	Unclassified virus	1	557	97	9.57E-34	38	RdRp
Southern tomato virus	*Amalgavirus*	1	867	70	5.72E-41	37	RdRp
Ourmia melon virus	*Ourmiavirus*	3	2079	58	7.15E-27	37	RdRp
Pepper cryptic virus 2	*Deltapartitivi rus*	1	615	95	1.25E-26	36	RdRp
Citrus leprosis virus C	*Cilevirus*	1	835	89	4.35E-21	35	RdRp
Apple mosaic virus	*Ilarvirus*	1	1910	20	3.37E-14	35	1a (VM)
Rhododendron virus A	*Amalgavirus*	1	599	45	1.71E-07	32	RdRp

Phaseolus vulgaris endornavirus 2	*Endornavirus*	1	4100	14	3.11E-20	32	PP (VH)
Rose cryptic virus 1	Unclassified virus	1	609	95	9.58E-20	32	RdRp
Fig cryptic virus	*Deltapartiti-virus*	1	522	87	2.84E-12	31	RdRp
Epirus cherry virus	*Ourmiavirus*	2	1334	54	1.43E-12	31	RdRp
Persea americana endornavirus 1	Unclassified virus	1	602	98	4.18E-26	31	PP (RdRp)
Grapevine leafroll-associated virus 2	*Closterovirus*	1	510	78	3.98E-07	28	RdRp
Beet virus Q	*Pomovirus*	1	9107	26	4.29E-39	28	VH, RdRp
Oryza sativa endornavirus	*Endornavirus*	1	1024	99	7.30E-11	27	PP
Vicia faba endornavirus	*Endornavirus*	1	599	83	2.87E-08	26	PP
Grapevine leafroll-associated virus 4	*Ampelovirus*	1	1022	84	8.34E-14	25	RdRp
**Island Grove**							
Blueberry mosaic associated virus	*Ophiovirus*	10	1680	100	0	97	23kDa, MP, NP, RdRp
Persimmon latent virus	Unclassified virus	5	4832	100	0	54	PArp, RdRp
Oryza rufipogon endornavirus	*Endornavirus*	1	898	34	2.15E-18	51	PP
Bell pepper endornavirus	*Endornavirus*	2	779	89	5.74E-32	37	PP (RdRp)
Raphanus sativus cryptic virus 2	Unclassified virus	1	537	88	1.49E-09	30	RdRp
Lagenaria siceraria endornavirus-California	*Endornavirus*	1	928	28	9.84E-06	29	PP

Fragaria chiloensis cryptic virus	Unclassified virus	2	699	92	7.16E-14	29	RdRp
Phaseolus vulgaris endornavirus 1	*Endornavirus*	1	1305	56	5.72E-13	27	PP
**Interlachen cultivated site**							
Blueberry latent virus	*Amalgavirus*	3	1116	100	0	100	CP, RdRp
Tobacco mosaic virus	*Tobamovirus*	7	3060	100	0	100	CP, MP, RdRp
Tomato spotted wilt virus	*Tospovirus*	1	521	74	7.90E-87	100	NP
Oryza sativa endornavirus	*Endornavirus*	2	7644	58	5.74E-114	40	PP, RdRp
Zucchini green mottle mosaic virus	*Tobamovirus*	1	696	88	4.67E-26	35	RdRp
Citrus leprosis virus C	*Cilevirus*	1	934	79	1.24E-23	32	RdRp
Sweet potato vein clearing virus	*Solendovirus*	1	555	99	3.63E-11	28	CP
Rice grassy stunt virus	*Tenuivirus*	1	766	96	7.69E-19	26	P1.339K
Phaseolus vulgaris endornavirus 1	*Endornavirus*	1	3537	28	4.34E-07	22	PP
**Island Grove cultivated site**							
Blueberry latent virus	*Amalgavirus*	2	2401	95	0	99	CP, RdRp
Blueberry mosaic associated virus	*Ophiovirus*	6	2429	100	0	96	23kDa, MP, NP, RdRp
Blueberry red ringspot virus	*Soymovirus*	1	8392	24	0	93	CP, HP, MP, RT, TA
Prunus virus T	*Tepovirus*	10	2504	100	0	86	CP, MP, RdRp
Vicia faba partitivirus 1	Unclassified virus	1	570	86	8.01E-52	55	RdRp

Supplementary files

Magnaporthe oryzae ourmia-like virus	Unclassified ourmiavirus	1	532	97	1.79E-19	37	RdRp

CP: Coat protein; FP: Fusion protein; HP: hypothetical protein; MP: Movement protein; NP: Nucleocapsid protein; P2: major core protein; PArp: proline-alanine-rich protein; PP: polyprotein; Rep: Replicase; RdRp: RNA-dependent RNA polymerase; RT: Reverse transcriptase; TA: Translational activator; VH: Viral helicase; VM: Viral methyltransferase. No. of hits correspond to the no. of scaffolds that produced sequence similarity to the closely related virus species by BLASTx.

Table S3. The number of SNPs identified from reads aligned to the assembled viral genomes in the corresponding viromes using FreeBayes in Geneious Prime v2019.1.3 with default parameter.

**Virus**	**HS**	**IL**	**ILC**	**IGC**
BlLV		**8	2	10
BlMaV	*24-278			***4-122
BRRV				74
BlVT				70

*RNA1: 278, RNA2: 58, RNA3: 24; **RNA3: 8; ***RNA1: 122, RNA2: 22, RNA3: 4; BlMaV: blueberry mosaic associated ophiovirus; BlLV: blueberry latent virus; BRRV: blueberry red ringspot virus; BlVT: blueberry virus T; HS: High Springs; IL: Interlachen; ILC, Interlachen cultivated site; IGC, Island Grove cultivated site.

Supplementary files

Figure S1. Total no. of identified plant virus scaffolds according to genera in the whole viromes of wild and cultivated *V. corymbosum* viromes.

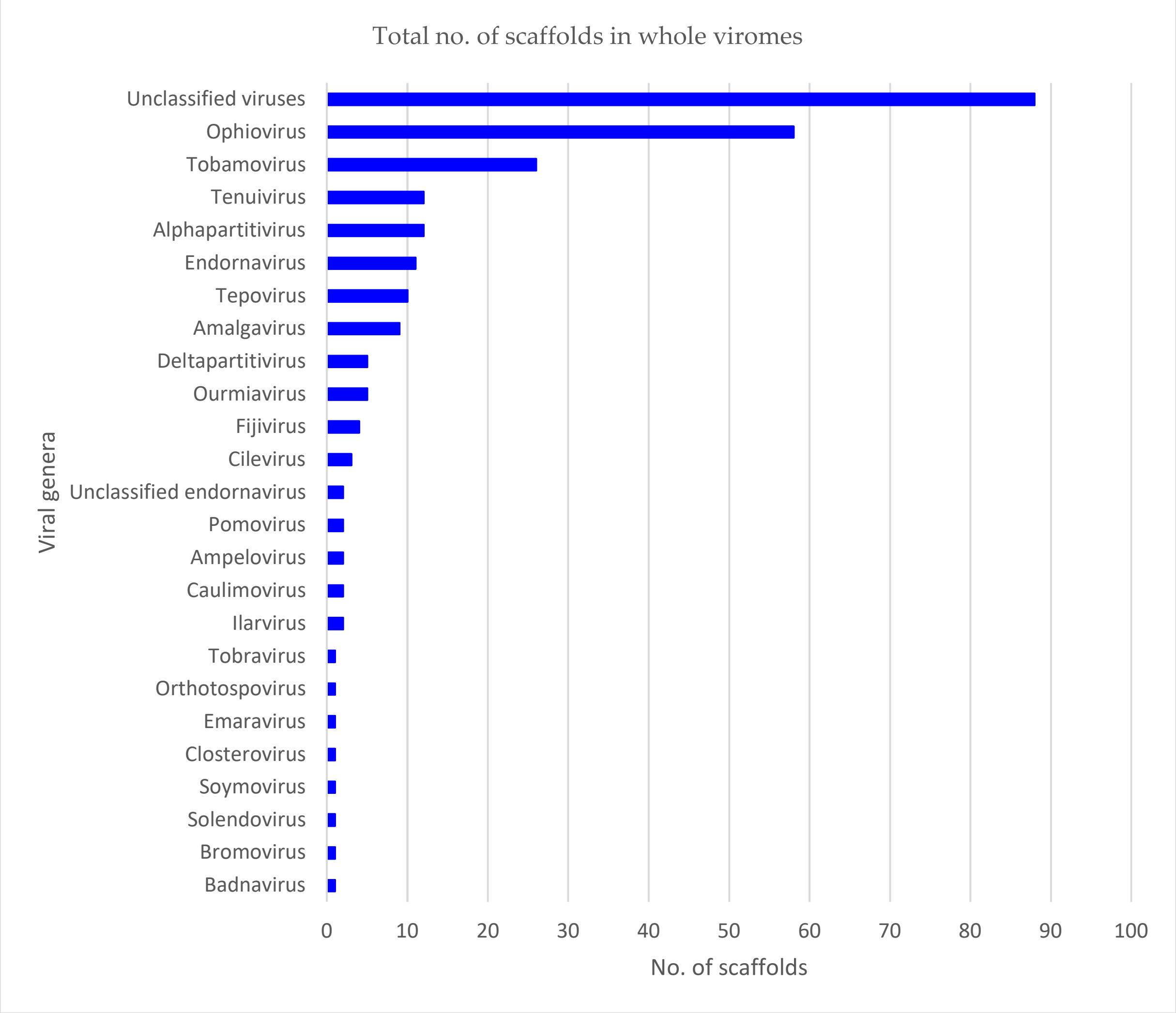

Supplementary files

Figure S2. Total no. of identified plant virus scaffolds according to genera in the viromes of wild *V. corymbosum* viromes.

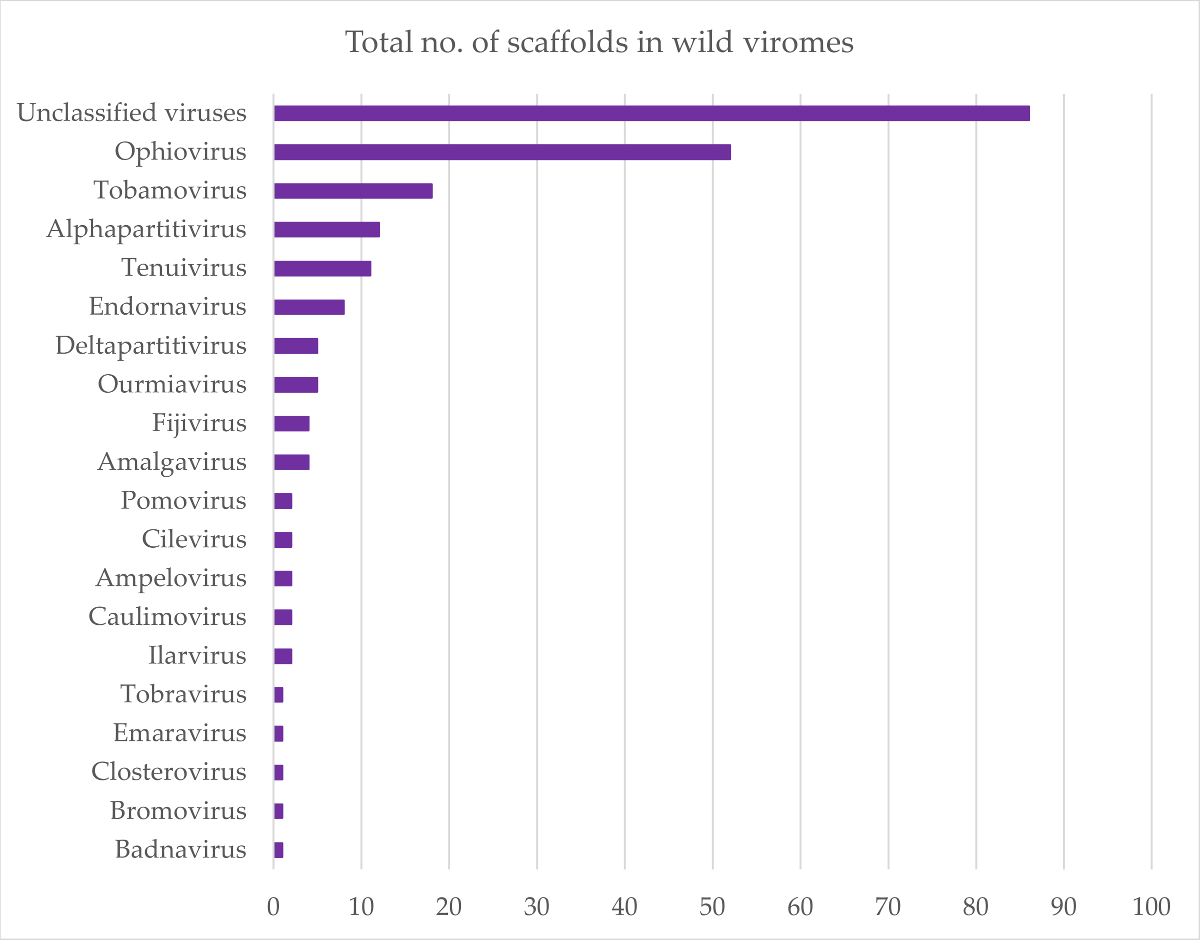

Supplementary files

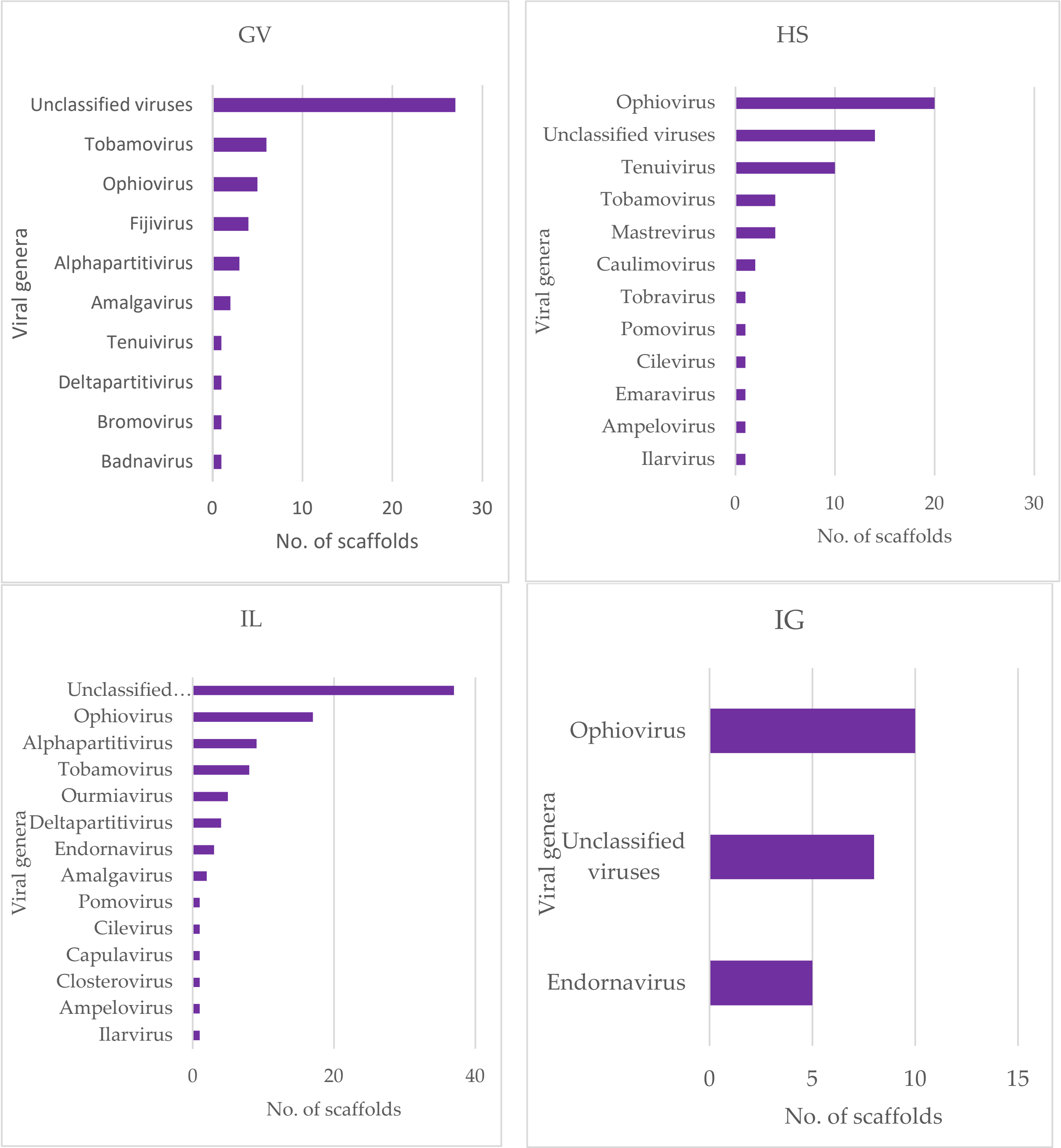

Supplementary files

Figure S3. Total no. of identified plant virus scaffolds according to genera in the viromes of cultivated *V. corymbosum* viromes.

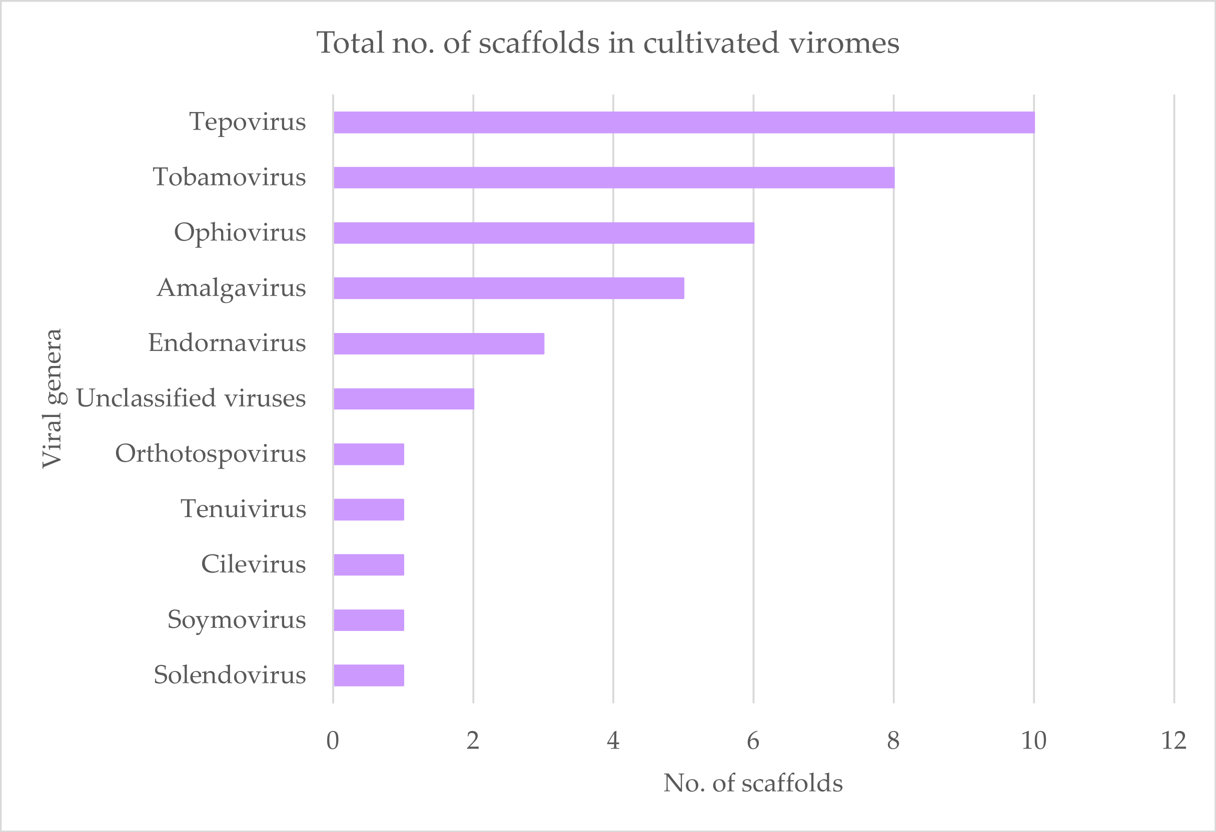

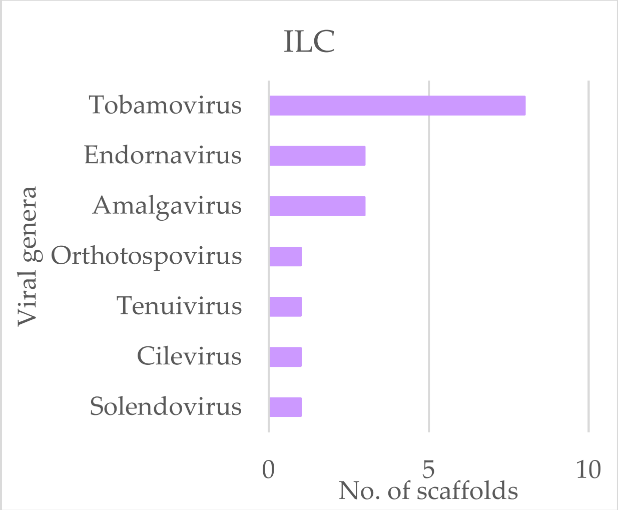

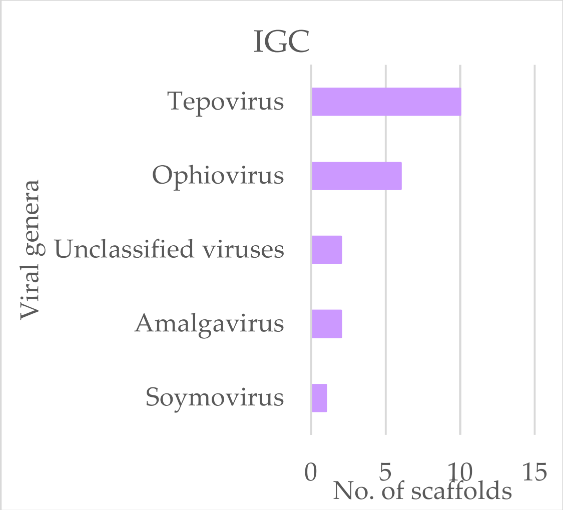

Supplement: Supplementary file 1 [file viruses-13-01165-s001.zip › viruses-1223629-supplementary.pdf]
